# Prebiotics Together with Raspberry Polyphenolic Extract Mitigate the Development of Nonalcoholic Fatty Liver Diseases in Zucker Rats

**DOI:** 10.3390/nu15143115

**Published:** 2023-07-12

**Authors:** Bartosz Fotschki, Michał Sójka, Monika Kosmala, Jerzy Juśkiewicz

**Affiliations:** 1 Division of Food Science, Institute of Animal Reproduction and Food Research, Tuwima 10, 10-748 Olsztyn, Poland; j.juskiewicz@pan.olsztyn.pl; 2 Institute of Food Technology and Analysis, Łódź University of Technology, Stefanowskiego 4/10, 90-924 Łódź, Poland; michal.sojka@p.lodz.pl (M.S.); monika.kosmala@p.lodz.pl (M.K.)

**Keywords:** ellagitannins, anthocyanins, prebiotics, raspberry polyphenolic extract, lipid metabolism, obesity, liver disorder, FXR receptor, AHR receptor, Zucker rat

## Abstract

Previous studies suggested that dietary supplementation with prebiotic fructooligosaccharides (FOSs) and polyphenols could mitigate disorders related to the first stage of nonalcoholic fatty liver disease (NAFLD) induced by an obesogenic diet. Therefore, this experiment aimed to address whether the health-promoting potential of raspberry polyphenols together with FOSs can regulate advanced-stage NAFLD in Zucker rats genetically predisposed to develop obesity. The addition of FOSs and raspberry polyphenolic extract to the diet reduced liver fat accumulation and triglyceride, free fatty acid, and total cholesterol levels in the liver. The elevated GSH/GSSG ratio and reduced malondialdehyde content indicated that the liver antioxidant potential was considerably increased. The treatment also lowered the plasma aminotransferase and alkaline phosphatase activities and collagen type IV levels. Insulin levels were decreased, but glucose levels remained constant, indicating greater insulin sensitivity. These changes may result from the upregulation of FXR and AHR receptors in the liver, which are responsible for regulating lipid metabolism and glucose and bile acid synthesis. The reduced bile acid levels in the cecal contents confirmed the activation of liver mechanisms. In conclusion, dietary enrichment with FOSs and raspberry polyphenolic extract has sufficient health-promoting potential to regulate liver metabolism, oxidative stress, and inflammation related to NAFLD development in obese Zucker rats.

## 1. Introduction

According to WHO reports, most people living in developing countries struggle with obesity-related disorders that affect multiple body systems [1]. There are two main factors responsible for this situation: The first is well-established environmental factors, such as poor eating habits, and the second is a genetic predisposition to develop obesity [2]. One of the disorders closely associated with the development of obesity is nonalcoholic fatty liver disease (NAFLD). It is associated with many features of metabolic syndrome and has become a very common liver pathology worldwide, affecting an estimated 25–30% of the population [3,4]. Currently, the pathophysiological model for NAFLD is the “multiple-hit” model [5]. Insulin resistance, adipose-derived hormones, dietary variables, the gut microbiome, and genetic and epigenetic factors are examples of such hits. The underlying mechanisms of NAFLD are associated with the accumulation of triacylglycerides (TAGs) and free fatty acids (FFAs) in the liver as a result of changes in the influx, synthesis, oxidation, and transport of fatty acids. Increasing fat accumulation in the liver disrupts many mechanisms that can lead to oxidative imbalance, insulin resistance, and the induction of inflammation as a result of mitochondrial dysfunction, lipid peroxidation, and the activation of inflammatory pathways [6,7]. For the treatment of NAFLD, a number of pharmaceutical approaches have been suggested, although the reported outcomes are inconsistent. Our previous studies showed that the initial changes in the functioning of the liver caused by nonalcoholic fatty liver (NAFL) might be effectively mitigated via supplementation with a combination of fructooligosaccharides (FOSs) and polyphenolic extract [8]. Nevertheless, these nutritional studies presented an incomplete picture of the abnormalities typical for the first stage of NAFL induced by an obesogenic diet. There is a lack of information on how and to what extent the dietary combination of FOSs and polyphenolic extract may affect the advanced stages of NAFLD.

Along with the expansion of NAFL, there is an increase in hepatocellular injury and the development of nonalcoholic steatohepatitis (NASH). Excessive fatty acids in the liver make the liver more vulnerable to metabolic disorders and injury. The main mechanisms responsible for the development of NASH originate from the mechanisms of liver peroxisomal fatty acid oxidation, the production of reactive oxygen species, disturbances in the metabolism of fatty acids, and inflammation that can lead to cirrhosis and hepatocellular carcinoma [5]. Furthermore, the involvement of the farnesoid X receptor (*fxr*) as a key molecular switch to regulate bile acid synthesis and thus the progression of NASH has also recently received attention [9,10]. The development of NASH is characterized by hepatocyte ballooning, cytoskeletal aggregation, apoptosis, and necrosis [11]. Insulin resistance is also a part of the second stage. Collagen deposition caused by the activation of hepatic stellate cells as well as portal fibrosis caused by ductular proliferation lead to the development and progression of NASH. These changes are often correlated with insulin resistance, which is now believed to cause the progression of steatosis to NASH and progressive fibrosis [12].

One of the supporting measures to counteract the progression of NAFL to NASH is a diet enriched with polyphenols. Raspberries (*Rubus idaeus*) are a valuable source of these bioactive compounds and are considered potent antioxidants. The anti-reactive species action is largely ascribed to the high content of ellagitannins and anthocyanins [13]. In addition to their powerful antioxidant activity, the aforementioned polyphenols are reported to possess anti-inflammatory properties, regulate liver metabolism, diminish the proliferation of human cancer cells, and benefit the blood lipid profile [14]. Nonetheless, the health-promoting potential of raspberry polyphenols has not been well investigated and evaluated. Our previous in vitro study on hepatocytes and in vivo study in Wistar rats showed that native polyphenols from raspberry alone regulate the immuno-metabolic signals associated with obesity and NAFL [8,15]. Furthermore, the dietary complex of raspberry polyphenols and prebiotics (FOSs) significantly enhanced the level of polyphenolic metabolites in the liver and thus enhanced the nutritional potential of polyphenols to regulate liver disorders [16]. The effect was linked to the stimulation of the intestinal microbiota to increase the production of polyphenolic metabolites and thus enhance the favorable effect of the polyphenolic extract on liver disorders in Wistar rats fed an obesogenic diet.

Based on the results of our own research, in this study, we aimed to answer the question of whether the increased health-promoting potential of raspberry polyphenol extract through the action of FOSs has sufficient potential to regulate the liver mechanisms responsible for the development of NAFLD in Zucker rats with a genetic predisposition toward obesity.

## 2. Materials and Methods

### 2.1. Raspberry Polyphenolic Extract Production

The frozen raspberry pomace of the “Polana” variety, an unused product from the manufacture of fruit juice, served as the source of the polyphenol extract. A detailed description of how the extract was prepared is provided in a previous study [8]. In brief, raspberry pomace was treated with a 30/70 (*v*/*v*) acetone–water mixture (Sigma-Aldrich, St. Louis, MO, USA). Three-step extraction resulted in the polyphenolic-containing mixture, which was subjected to a filtration procedure with the aid of a Hobrafit S40N cellulose filter with 5 μm nominal retention (Hobra-Školnik S.R.O., Broumov, Czech Republic). The next stage was to remove the acetone from the extraction mixture with an evaporator (Heidolph Hei-Vap) combined with an Automatic Hei-Vap Distimatic Module (Heidolph, Schwabach, Germany). The following temperature and pressure conditions were used: 60 °C and 135 mbar. An additional filtration process with a Hobrafit S40N filter was carried out to obtain the acetone-free extract. The extract purification procedure was conducted with the aid of an Amberlite XAD 1600 sorption bed in conjunction with a column that measured 50 cm in length and 7.5 cm in diameter. Gravity was used to carry out the purification process, and the liquid flow rate was between 20 and 40 mL/min. Polyphenols were eluted with 30% and 40% ethanol (POCH, Gliwice, Poland). Formic acid (Sigma-Aldrich, St. Louis, MO, USA) was added to all elution solutions to reach an acidity of 0.01%. The final step was evaporating the ethanol using a Heidolph Hei-Vap Distimatic evaporator at 60 °C and 125 mbar and freeze-drying for 48 h at −36 °C to obtain the final product as a red powder. 

### 2.2. Chemical Characterization and Polyphenolic Analysis

The following procedures were used in accordance with AOAC methodologies to determine the preparations’ chemical composition: dry matter and ash content, 940.26; protein content, 920.152; crude fat, 930.09; and total dietary fiber, 985.29.

Following the method presented in an earlier article, the resulting extract’s ellagitannin and anthocyanin analyses were carried out [8]. Briefly, the polyphenolic extract was diluted with methanol (40 mg/10 mL for ellagitannins and 75 mg/10 mL for anthocyanins) and measured using a Knauer Smartline HPLC system with a DAD detector. For ellagitannin analysis, a Gemini C18 column (250 × 4.6 mm; 5 μm, Phenomenex, Torrance, CA, USA) was used, whereas for anthocyanins, a Gemini C18 column (150 × 4.6; 5 μm, Phenomenex) was used. Ellagitannin levels were detected at 250 nm, and the concentration of each respective compound was determined using the standard curves prepared for the internal standard, i.e., ellagic acid (Extrasynthese, Genay, France). A procedure established by our research group, which was developed at the Lodz University of Technology according to Klewicka et al. [17], was applied to obtain pure standards of sanguiin H-6 and lambertianin C. The quantity of each anthocyanin was calculated from the plot generated for cyanidin-3-O-glucoside (Extrasynthese, Genay, France) upon anthocyanin identification at 520 nm. Employing an LC-MS instrument (Q Exactive Orbitrap, Thermo Fisher Scientific, San Jose, CA, USA), the identification of ellagitannin and anthocyanin was carried out.

The proanthocyanidin analysis was performed according to a previous method described in a study by Sójka et al. [18]. Similar analysis conditions were applied, but a different liquid-chromatography equipment was used (Shimadzu, Tokyo, Japan), consisting of a Shimadzu LC-20AD Prominence HPLC Pump with maximum discharge pressure 40 MPa, a Shimadzu DGU-20A 5 Prominence Degasser with a fluoroethylene membrane, a Shimadzu CTO-10AS thermostat with the temperature regulation from 4 °C to 80 °C, and a SIL-20AC autosampler. The prepared curves of standard (−)-epicatechin (E1753-1G Sigma-Aldrich, USA) and (+)-catechin (C1251-10G Sigma-Aldrich, USA) as well as (−)-epicatechin–phloroglucinol adduct, for the terminal and extender units, respectively, were used in calculations of proanthocyanidin level in the extract. At the same time, similar to ellagitannin detection, HPLC analyses were carried out for free (−)-epicatechin and (+)-catechin. 

### 2.3. Animals and Experimental Design

The animal protocol used in the present study was approved by the local Institutional Animal Care and Use Committee (Committee Permission No. 29/2021; Olsztyn, Poland). The experiment was conducted with sixteen male Zucker rats (Crl:Zuc(Orl)-Lepr) aged eight weeks that were randomly allocated into two groups of eight animals each. These rats are a well-known animal model of genetic obesity caused by a mutation (fa) in the gene encoding the receptor. The average initial body weight for all rats was 188.2 g, with a standard deviation equal ±2.1 g. All animals were housed individually over 4 weeks in metabolic cages (Tecniplast Spa, Buguggiate, Italy) with free access to water and semipurified casein diets. To induce the advanced stage of NAFLD, high-fat diets were used. The diet intake was monitored at daily intervals. The environment was controlled with a 12 h light and 12 h dark cycle, a temperature of 21 ± 1 °C, and twenty air changes/h. The control diet contained cellulose as a fiber source (group C), and to enhance the progression of NAFLD, 23% lard was added to the diet, whereas in the experimental diet group, the dietary composition from the C group was enriched with FOSs (3%) and raspberry polyphenolic extract (0.63%) at the expense of cellulose and some maize starch (group CFP). To the experimental diet, 0.3% polyphenols were added. According to the results from previous studies, this amount of raspberry polyphenols and their combination with FOSs showed the most favorable effect against the first stage of NAFL in Wistar rats [15,19]. The diet compositions are shown in Table 1.

### 2.4. Collection of Biological Material and Analyses of Bile Acids in Cecum

The rats were carefully individually monitored for feed intake (daily) and body weight (weekly). At the termination of the study, the rats were anesthetized with a mixture of ketamine and xylazine (100 mg and 10 mg/kg BW, respectively) in 0.9% NaCl according to the recommendations for anesthesia of laboratory rats. The content of the fat tissue of anesthetized rats was determined using a time-domain nuclear magnetic resonance methodology with the aid of a Minispec LF90II analyzer (Bruker). The formula for the calculation of rat body fat percentage was as follows: body fat % = body [100%] − body lean tissue % − body fluids %. After abdominal dissection, blood was drawn into heparinized tubes from the caudal vena cava to obtain plasma. The collected blood was slowly centrifuged at a speed of 350× *g* for 10 min and at 4 °C. The obtained plasma samples were kept frozen at −80 °C until needed. Next, the cecal digesta were collected, and the livers were removed, weighed, frozen using liquid nitrogen, and stored at −80 °C until assayed. The total bile acids were analyzed using a commercial BA assay kit (Crystal Chem Inc., Chicago, IL, USA).

### 2.5. Plasma and Liver Markers of the Lipid Profile, Oxidative Stress, Inflammation, and Fibrosis

The plasma concentrations of total cholesterol, LDL cholesterol, HDL cholesterol, and triglycerides, as well as the plasma activities of aspartate transaminase (AST), alanine transaminase (ALT), alkaline phosphatase (ALP), albumin, and glucose, were determined using an automatic biochemical analyzer (Pentra C200, Horiba, Tokyo, Japan). To measure the concentration of free fatty acids in the liver and plasma, a commercial quantitation kit (Sigma-Aldrich, St. Louis, MO, USA) was used. The collagen type IV in plasma was measured with an enzyme-linked immunosorbent assay kit (Cloud-Clone Corp. Katy, TX, USA). The insulin level in plasma was determined using a commercial ELISA kit (Demeditec Diagnostics GmbH, Kiel, Germany). After the storage of the liver, the reduced glutathione (GSH) and oxidized glutathione (GSSG) concentrations were determined using an enzymatic recycling method described by Rahman et al. [20]. Malondialdehyde (MDA) was measured in liver tissue using a method established by Botsoglou et al. [21]. The MDA content was determined spectrophotometrically at 532 nm and expressed in micrograms of MDA per gram of liver tissue. The method developed by Folch et al. [22] was used to extract liver lipids. The levels of cholesterol and triglycerides in the isolated lipid phase of the liver were measured spectrophotometrically using commercial kits (Cholesterol DST and Tri-glycerides DST, Alpha Diagnostics, Ltd., San Antonio, TX, USA). The measurement of liver hydroxyproline levels as a proxy for the collagen content was prepared according to the description of a previous study [23]. Briefly, liver samples were homogenized in 2 ml of phosphate-buffered saline (Sigma-Aldrich, St. Louis, MO, USA) and stored at 4 °C overnight. The next day, 0.5 mL aliquots were hydrolyzed with 0.25 mL of 6 N HCl for 4.5 h at 120 °C. To construct a standard curve, hydroxyproline concentrations from 0 to 20 μg/mL were used (Sigma-Aldrich by Merck). Twenty microliters of each sample, defined as a standard curve point, was added to a 96-well plate and incubated for 20 min at room temperature with 50 μL of chloramine T solution (282 mg of chloramine T, 2 mL of n-propanol, 2 mL of distilled water, and 16 ml of citrate acetate buffer (5% citric acid, 7.24% sodium acetate, 3.4% sodium hydroxide, and 1.2% glacial acetic acid)). Next, 50 μL of Ehrlich’s solution (2.5 g of 4-(dimethylamino) benzaldehyde, 9.3 mL of n-propanol, and 3.9 ml of 70% perchloric acid) was added, and the plate was incubated for 15 min at 65 °C. After cooling the samples, the plate was read at 550 nm on a microplate reader (Multiskan Sky Microplate Spectrophotometer, Thermo Fisher Scientific™).

### 2.6. Plasma Lipid Profile and Inflammatory Markers

Within the blood plasma, the following compounds were estimated using a biochemical analyzer (Pentra C200, Horiba, Tokyo, Japan): TGs, TC, and fractions of high-density lipoprotein (HDL) cholesterol and the ALP, AST, and ALT activity.

### 2.7. RNA Isolation and Quantitative RT-PCR

Following the manufacturer’s guidelines, total RNA was isolated from liver tissues using TRIzol Reagent (Thermo Fisher Scientific, Waltham, MA, USA). On a NanoDrop 1000 (Thermo Fisher Scientific, Waltham, MA, USA), the concentration of RNA was confirmed. cDNA was synthesized from 500 ng of total RNA using a High-Capacity cDNA Reverse Transcription Kit with RNase Inhibitor (Thermo Fisher Scientific, Waltham, MA, USA). *β-actin* was selected as a reference gene. The mRNA expression levels of aryl hydrocarbon receptor (*ahr*), cholesterol 7α-hydroxylase (*cyp7a1*), 25-hydroxycholesterol 7α-hydroxylase (*cyp7b1*), hypoxia-inducible factor 1α (*hif1α*), peroxisome proliferator-activated receptor alpha (*pparα*), peroxisome proliferator-activated receptor gamma (*pparγ*), sterol regulatory element-binding protein 1 (*srebp1c*), nuclear receptor farnesoid X receptor (*fxr*), and small heterodimer partner (*shp*) were assessed using single-tube TaqMan^®^ Gene Expression Assays (Life Technologies, MA, USA). Amplification was performed using a 7900HT Fast Real-Time PCR System. The mRNA expression levels were normalized to the reference gene *β-actin*.

### 2.8. Liver Histopathology

The preparation of liver samples and analyses were performed according to a previously described method [15]. Briefly, formalin-fixed and paraffin-embedded liver samples (1–2 μm thick) were stained with hematoxylin and eosin (H&E; Merck, Darmstadt, Germany). The H&E-stained sections (*n* = 8 animals per group) were visualized using standard light microscopy with an Olympus microscope (BX43) (Olympus Co., Tokyo, Japan) equipped with an Olympus digital camera (XC50). These criteria were used to assess hepatic steatosis: grade 0, no fat; grade 1, steatosis occupying less than 33% of the hepatic parenchyma; grade 2, 34–66% of the hepatic parenchyma; and grade 3, more than 66% of the hepatic parenchyma. For inflammatory cell infiltration, the evaluation was as follows: grade 0, none; grade 1, 1–2 foci/field; grade 2, 3–4 foci/field; and grade 3, more than 4 foci/field [24]. Ballooning was graded as none, minimal, mild, or marked [25].

### 2.9. Statistical Analysis

The results were analyzed statistically using Student’s *t*-test. The Shapiro–Wilk test was used to test the normal distribution. The results are expressed as the mean value and standard error of the mean (SEM, *n* = 8). Calculations were performed using STATISTICA 8.0 (StatSoft Corporation, Krakow, Poland).

## 3. Results

The chemical characterization and profile of polyphenols in the raspberry polyphenolic extract are presented in Table 2. The examined polyphenolic extract contained 47.80 ± 1.06 g/100 g of polyphenols. The analysis of the polyphenolic profile showed that ellagitannins are the most common in the preparation, mainly sanguiin H-6 and lambertianin C. There were also flavanols, primarily proanthocyanidins as well as anthocyanins, mainly cyanidin-3-O-spohoroside and cyanidin-3-O-glucoside.

**Figure 1:**
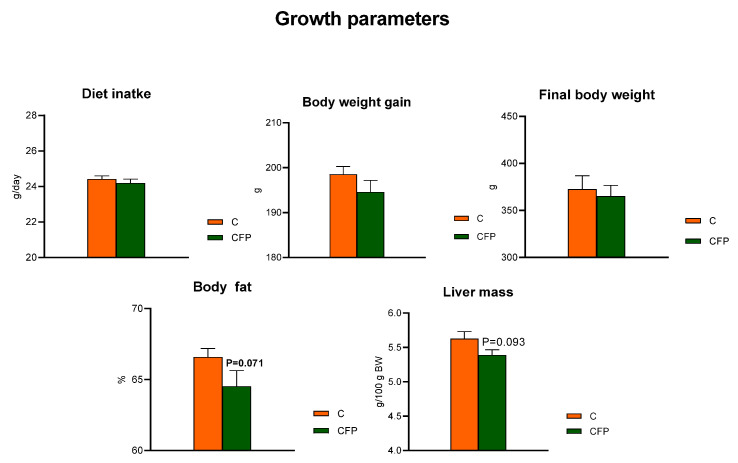
Growth parameters of Zucker rats fed the experimental diet. The results are presented as the mean ± SEM (standard error of the mean), *n* = 8. C, standard diet for laboratory rodents enriched with lard; CFP, diet C supplemented with fructooligosaccharides and raspberry polyphenolic extract. BW, body weight.

The *t*-test showed that the dietary application of raspberry polyphenolic extract combined with FOSs (CFP treatment) did not affect the diet intake compared with that of the C group, whose diet was not supplemented with the extract and FOSs (*p* > 0.05; Figure 1). The measured body weight gain (BWG) during the feeding period and the final body weight of rats did not differ between the C and CFP groups (*p* > 0.05). The relative liver mass of rats fed the CFP diet tended to be decreased (*p* = 0.093) in relation to that of the control animals. Additionally, the nuclear magnetic resonance analysis conducted on living animals on the final experimental day showed a tendency toward lowered body fat percentage in the CFP rats compared with the C group *(p* = 0.071).

**Figure 2:**
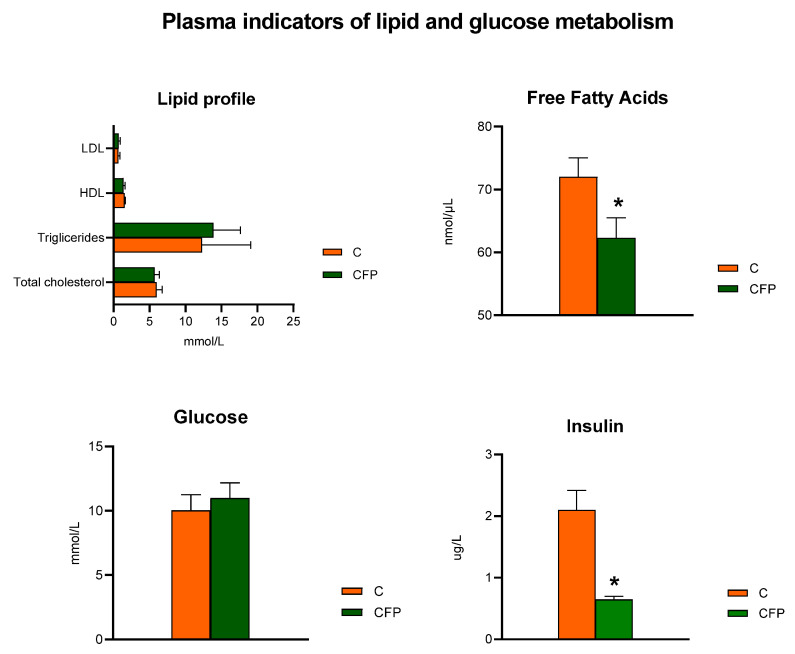
Plasma indicators of lipid and glucose metabolism in Zucker rats fed the experimental diet. The results are presented as the mean ± SEM (standard error of the mean), *n* = 8. C, standard diet for laboratory rodents enriched with lard; CFP, diet C supplemented with fructooligosaccharides and raspberry polyphenolic extract. HDL, HDL cholesterol, LDL, LDL cholesterol. * Data are significantly different from the C group (*p* ≤ 0.05, *t*-test).

The concentrations of low-density lipoprotein (LDL) and HDL fractions in the blood plasma were comparable (*p* > 0.05) in rats fed the C and CFP diets (Figure 2). The within-group variability was relatively high, resulting in plasma triglyceride concentrations that were not significantly different between the C and CFP groups (*p* > 0.05). The dietary addition of the raspberry extract and FOSs did not change the total cholesterol concentration in rat plasma in comparison to that of the control C animals. The experimental treatment with the extract and FOSs resulted in a significant decrease in free fatty acids as well as insulin concentrations in the plasma of rats compared with those of the control group (*p* < 0.05 in both cases). Interestingly, the blood plasma glucose concentration did not differ significantly between the C and CFP groups (*p* > 0.05).

**Figure 3:**
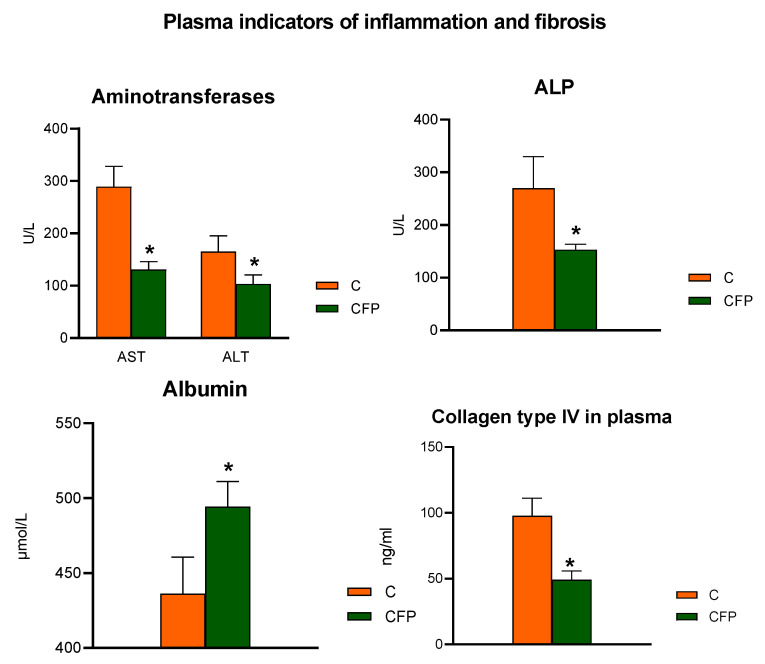
Plasma indicators of inflammation and fibrosis in Zucker rats fed the experimental diet. The results are presented as the mean ± SEM (standard error of the mean), *n* = 8. C, standard diet for laboratory rodents enriched with lard; CFP, diet C supplemented with fructooligosaccharides and raspberry polyphenolic extract. AST, aspartate transaminase; ALT, alanine transaminase; ALP, alkaline phosphatase. * Data are significantly different from the C group (*p* ≤ 0.05, *t*-test).

Figure 3 shows the data regarding blood plasma inflammatory and fibrosis parameters in Zucker rats fed experimental diets. The plasma activities of AST, ALT, and ALP were significantly diminished in the rats subjected to the CFP diet compared with the control C animals (*p* < 0.05). An increase in the concentration of plasma albumin was observed following dietary treatment with FOSs and raspberry polyphenolic extract (CFP > C; *p* < 0.05). The CFP group had a significantly lower concentration of collagen type IV in plasma than that in the C group (*p* < 0.05).

**Figure 4:**
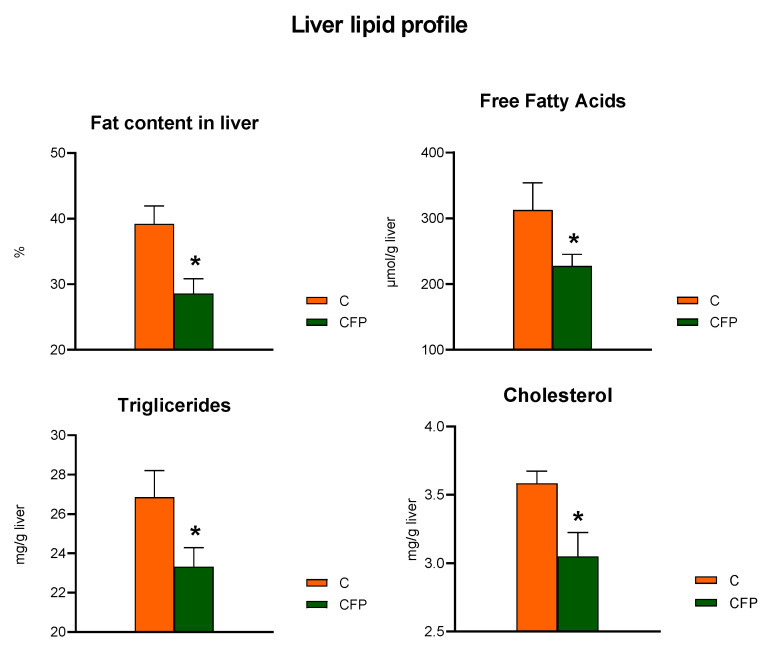
Liver indicators of lipid metabolism in Zucker rats fed the experimental diet. The results are presented as the mean ± SEM (standard error of the mean), *n* = 8. C, standard diet for laboratory rodents enriched with lard; CFP, diet C supplemented with fructooligosaccharides and raspberry polyphenolic extract. * Data are significantly different from the C group (*p* ≤ 0.05, *t*-test).

**Figure 5:**
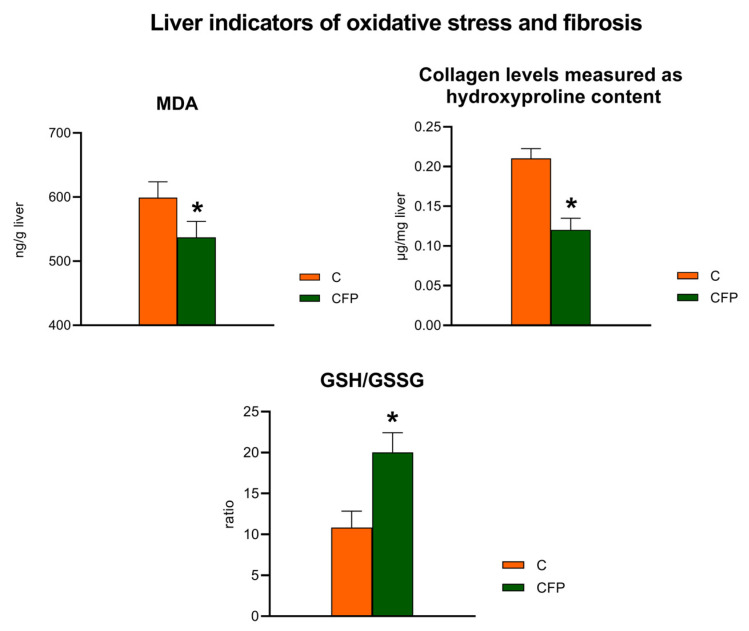
Liver indicators of oxidative stress and fibrosis in Zucker rats fed the experimental diet. The results are presented as the mean ± SEM (standard error of the mean), *n* = 8. C, standard diet for laboratory rodents enriched with lard; CFP, diet C supplemented with fructooligosaccharides and raspberry polyphenolic extract. MDA, malondialdehyde; GSH/GSSG, ratio of reduced glutathione (GSH) to oxidized glutathione (GSSG). * Data are significantly different from the C group (*p* ≤ 0.05, *t*-test).

The fat concentration in the liver of the CFP group was significantly decreased in comparison to that in the control rats (*p* < 0.05; Figure 4). Additionally, decreased concentrations of hepatic free fatty acids, triglycerides, and total cholesterol were observed following the dietary application of the raspberry extract coupled with FOSs. The liver concentrations of MDA and collagen, the latter measured as the hydroxyproline content, were significantly decreased in the CFP group compared with the C group (*p* < 0.05; Figure 5). The liver of the rats fed a diet supplemented with the raspberry extract and FOSs was characterized by an elevated ratio of GSH to GSSG in comparison to the ratio in the C group (*p* < 0.05 vs. C).

**Figure 6:**
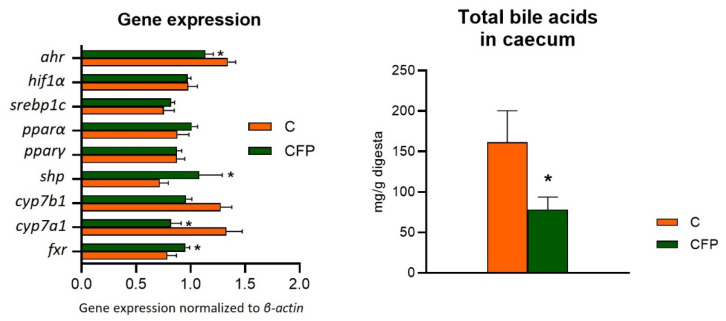
Selected liver molecular factors of oxidative stress, lipid metabolism, inflammation, and bile acid levels in the cecum of Zucker rats fed the experimental diet. The results are presented as the mean ± SEM (standard error of the mean), *n* = 8. C, standard diet for laboratory rodents enriched with lard; CFP, diet C supplemented with fructooligosaccharides and raspberry polyphenolic extract. *ahr*, aryl hydrocarbon receptor; *cyp7a1*, cholesterol 7α-hydroxylase; *cyp7b1*, 25-hydroxycholesterol 7α-hydroxylase; *hif1α*, hypoxia-inducible factor 1α; *pparα*, peroxisome proliferator-activated receptor alpha; *pparγ*, peroxisome proliferator-activated receptor gamma; *srebp1c*, sterol regulatory element-binding protein 1; *fxr*, farnesoid X receptor; *shp*, small heterodimer partner. * Data are significantly different from the C group (*p* ≤ 0.05, *t*-test).

Figure 6 presents data regarding selected hepatic molecular factors describing the oxidative status, lipid metabolism, inflammation processes, and bile acid levels in the cecum of Zucker rats fed experimental diets. The measured differences between the CFP vs. C groups for the relative expression of hypoxia-inducible factor 1α (*hif1α*), sterol regulatory element-binding protein 1c (*srebp1c*), and peroxisome proliferator-activated receptors alpha and gamma (*pparα* and *pparγ*, respectively) did not reach statistical significance (*p* > 0.05). In contrast, the relative expression (normalized to *β-actin*) of *ahr*, 25-hydroxycholesterol 7-alpha-hydroxylase, and cholesterol 7 alpha-hydroxylase (*cyp7b1* and *cyp7a1*; cytochrome P450 family 7 subfamily B member 1 and cytochrome P450 family 7 subfamily A member 1, respectively) was significantly decreased in the livers of rats fed a diet with the raspberry extract and fructooligosaccharides (*p* < 0.05 vs. C). The CFP group had enhanced hepatic expression of the *shp* and *fxr* genes compared with expression in the C group. Moreover, the GFP group had considerably higher levels of bile acids in the cecum digesta (*p* < 0.05 vs. C).

**Figure 7:**
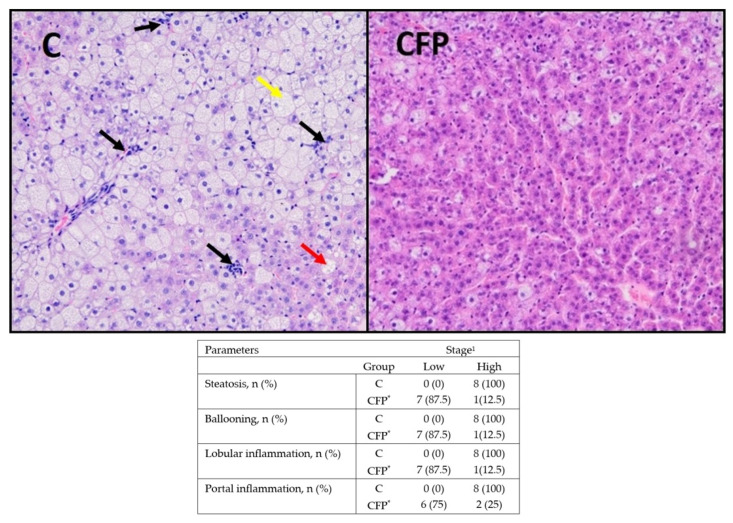
Effect of the experimental diet on Zucker rats during the development of NAFLD (*n* = 8). C, standard diet for laboratory rodents enriched with lard; CFP, diet C supplemented with fructooligosaccharides and raspberry polyphenolic extract. The histological preparations from the C group show a site of inflammation within the hepatic lobule (black arrow), enhanced hepatocyte ballooning (yellow arrow), and one of many lipid droplets (red arrow). The histological preparations from the CFP group showed a reduced amount of hepatocyte ballooning, lipid droplets, and hepatocytes with rounded contours with clear reticular cytoplasm. The H&E-stained liver section is presented at 20× magnification. The results in the table are presented as the number of n with a low or high stage of selected liver parameters and the percentage of n in the group. * Data in the table are significantly different from the C group (*p* ≤ 0.05, *t*-test). ^1^ Low = stage 0 or 1; High = stage 2 or 3.

The histological analyses of the liver in Zucker rats showed that disorders related to the development of NAFLD might be reduced when the diet is enriched with fructooligosaccharides and raspberry polyphenolic extract (Figure 7). In this group, a significant reduction in the development of liver steatosis and portal and lobular inflammation was observed; moreover, hepatocyte ballooning and lipid droplet accumulation were also considerably reduced (*p* < 0.05 vs. C).

## 4. Discussion

NAFLD has become the most common liver disease worldwide. This serious chronic liver condition can progress to cirrhosis, liver cancer, and death. NAFLD is pathologically characterized as NAFL, and when the liver accumulates fat and the ballooning of hepatocytes progresses, the condition might transform into NASH [26]. In this study, to increase the extent of disorders related to NAFLD for analysis, Zucker rats with a genetic predisposition to develop obesity were used [27]. This animal model partially simulates human metabolic syndrome through the accumulation of fat and triglycerides and the development of oxidative stress in the liver, insulin resistance, mild hyperglycemia, and hyperlipidemia.

Our previous studies on Wistar rats fed a high-fat diet showed that raspberry polyphenolic extract combined with FOSs considerably enhanced the health-promoting potential against disorders related to increased liver fat accumulation [15]. This effect is related to the elevated metabolism of polyphenols by the intestinal microbiota to chemical forms with high bioactivity and bioavailability and thus enhanced regulatory effects in the liver [16]. In this study, the health-promoting potential of polyphenolic extract and FOSs was strong enough to regulate liver disorders in Zucker rats. Lower concentrations of fat, free fatty acids, triglycerides, cholesterol, hepatocyte ballooning, and steatosis in the liver were observed. Other studies have shown that the considerable influence on mechanisms related to disorders in liver lipid metabolism is associated with changes in the expression of *srebp1c*, *pparα*, and *pparγ* [27,28]. To regulate metabolic disorders, these genes are often selected as targets for many kinds of polyphenols, including those from raspberries [15,29]. Furthermore, supplementation with FOSs alone may have an impact on hepatic *pparγ* expression through the stimulation of the intestinal bacteria to produce short-chain fatty acids, which can act as ligands for this receptor and mitigate disorders related to NAFL [30]. Surprisingly, in this study, these genes were not altered. However, the combination of FOSs and polyphenolic extract considerably elevated the liver expression of *fxr*. This receptor regulates the mechanisms associated with liver lipid metabolism and bile acid synthesis, in which increased levels of bile acid activate *fxr*, causing the suppression of bile acid synthesis through the activation of *shp* [9,10]. This mechanism leads to the activation of liver cytochromes, e.g., *cyp7a1* and *cyp7b1*, which play a main role in the transformation of cholesterol to bile acids and thus the regulation of lipid metabolism in the liver [10]. In our study, the upregulation of *shp* and downregulation of *cyp7a1* and *cyp7b1* were observed in the liver of Zucker rats fed a diet enriched with FOSs and polyphenolic extract. The activation of this mechanism considerably lowered the level of bile acids in the cecum and thus favorably affected lipid metabolism in the liver and body fat accumulation.

Stofan et al. (2020) [10] noted that the disruption of bile acid homeostasis often leads to liver disorders, including cholestasis, hepatic steatosis, and fibrosis. In this study, a diet with FOSs and polyphenolic extract activated the *fxr*-related mechanisms responsible for the regulation of bile acid synthesis and reduced the levels of collagen type IV in the plasma and collagen in the liver, which are markers of liver fibrosis development [10,31]. Furthermore, studies on mice and humans have shown that *fxr* activation results in a range of therapeutically significant changes, including lower hepatic triglyceride levels, decreased inflammation, and enhanced insulin sensitivity [32]. A similar effect was observed in our nutritional study with Zucker rats. Supplementation with FOSs and polyphenolic extract considerably regulated markers of the development of liver inflammation, e.g., it decreased the activity of aminotransferases and alkaline phosphatase in the plasma and the stage of portal and lobular inflammation in the liver. In addition, in rats fed a diet with FOSs and polyphenolic extract, reduced insulin levels were noted with unchanged glucose levels. These changes may indicate the regulation of the mechanisms responsible for insulin sensitivity in Zucker rats. Another important parameter associated with the development of NASH is a lower level of albumin in the blood [33]. The biological and therapeutic role of this protein in liver disease is related to oncotic effects, immunomodulation, antioxidant effects, and the binding of multiple molecules, including polyphenols [34]. In this study, FOSs together with polyphenolic extract considerably increased albumin levels in the plasma (Figure 3). This favorable effect might also be linked with the activation of liver *fxr*, which is involved in the activation of mechanisms that oppose the loss of albumin in the blood during the development of NASH [35].

In addition to the upregulation of *fxr* in the liver, elevated expression levels of the multifunctional receptor *ahr* were also observed. According to an earlier study, where a diet with FOSs and polyphenols increased the number of polyphenol metabolites in the liver [16], it might be assumed that dietary polyphenolic compounds such as *ahr*-targeted compounds might affect the expression level of this receptor. Some studies have described the pharmacological/therapeutic use of polyphenols as *ahr* antagonists [36]. In their study on C57BL/6J mice and HepG2 cells, Zhu et al. (2020) [37] highlighted the important role of liver *ahr* in the suppression of fatty acid oxidation and the inhibition of the hepatic export of triglycerides. *ahr* is also involved in the mechanism regulating liver oxidative stress [38]. Therefore, the upregulation of this receptor might partially explain the favorable effect of FOSs and polyphenolic extract on markers of oxidative stress in the liver of Zucker rats. A reduction in the malondialdehyde content and elevation of the GSH/GSSG ratio in the liver were observed.

## 5. Conclusions

Dietary supplementation with FOSs and raspberry polyphenolic extract regulates the disorders related to advanced-stage NAFLD in Zucker rats with a genetic predisposition toward obesity. This favorable impact is probably linked with the activation of the liver FXR-related mechanism responsible for bile acid homeostasis and the multifunctional liver receptor AhR. The activation of these mechanisms might explain the observed mitigation of disorders in lipids, glucose metabolism, antioxidant status, and inflammation as well as the reduction in indicators of liver fibrosis development. Based on the results, the examined combination of prebiotic FOSs and raspberry polyphenolic extract has solid health-promoting potential to support the treatment of disorders associated with the advanced stage of NAFLD. Nevertheless, there are differences between animal models and humans in bile acid synthesis as well as in the metabolism of raspberry polyphenols by the intestinal microbiota; therefore, the use of FOSs and raspberry polyphenolic extract as functional additives to the diet should also be verified in human studies.

## Figures and Tables

**Table 1:** Diet compositions (%).

Ingredient	Group	
	C	CFP
Casein	20	20
DL-Methionine	0.3	0.3
Rapeseed oil	2	2
Lard	23	23
Fructooligosaccharides	--	3
Polyphenolic extract	--	0.63
Cellulose ^1^	5	2
Saccharose	10	10
Maize starch	35	31.63
Mineral mix ^2^	3.5	3.5
Vitamin mix ^2^	1	1
Choline chloride	0.2	0.2
Calculated content		
Fiber	5	5
Polyphenols	--	0.3
Protein	20	20

^1^ The α-cellulose preparation was obtained from Sigma-Aldrich (No. C8002). ^2^ AIN-93M (Reeves 1997).

**Table 2:** Basic chemical and polyphenolic composition of the raspberry polyphenolic extract.

Compound	
Basic chemical composition (*n* = 3), g/100 g	
Dry matter (AOAC 940.26)	94.79 ± 0.18
Protein (AOAC 920.152)	4.74 ± 0.45
Fat (AOAC 930.09)	0.51 ± 0.04
Ash (AOAC 940.26)	2.11 ± 0.06
TDF (AOAC 985.29)	0.00 ± 0.00
Total polyphenols	47.80 ± 1.06
Polyphenols (*n* = 3), mg/100 g	
Total polyphenols	47,804.8 ± 1060.5
Ellagitannins	
Sanguiin H-6	17,553.7± 352.4
Sanguiin H-10 ^a^	1300.6± 17.0
Lambertianin C	19,701.1± 1212.6
Ellagic acid	196.7± 18.1
Total ellagitannins	38,923.6 ± 1547.0
Flavanols	
(+)-Catechin	208.0 ± 5.4
(−)-Epicatechin	343.5 ± 5.5
Proanthocyanidins	7820.2 ± 475.7
Total flavanols	8371.7 ± 486.5
Anthocyanins ^b^	
Cyanidin-3-O-spohoroside	314.6 ± 11.5
Cyanidin-3-O-glucosyl-rutinoside	27.0 ± 0.7
Cyanidin-3-O-glucoside	152.0 ± 0.9
Cyanidin-3-O-rutinoside	11.5 ± 0.1
Pelargonidin-3-O-glucoside	4.5 ± 0.3
Total anthocyanins	509.6 ± 10.9

Values are expressed as the mean ± standard deviation (mg/100 g); *n*, number of measurements. TDF, total dietary fiber. Data were shown in a previous study [8]. ^a^ The contents of these substances were calculated based on the sanguiin H-6 standard. ^b^ The contents of anthocyanins were calculated based on the cyanidin-3-O-glucoside standard.

## Data Availability

Not applicable.

## Abbreviations

*ahr*, aryl hydrocarbon receptor; ALT, alanine transaminase; ALP, alkaline phosphatase; AST, aspartate transaminase; *cyp7a1*, cholesterol 7α-hydroxylase; *cyp7b1*, 25-hydroxycholesterol 7α-hydroxylase; *fxr*, farnesoid X receptor; GSH/GSSG, ratio of reduced glutathione (GSH) to oxidized glutathione (GSSG); *hif1α*, hypoxia-inducible factor 1α; HDL, HDL cholesterol; LDL, LDL cholesterol; MDA, malondialdehyde; *pparα*, peroxisome proliferator-activated receptor alpha; *pparγ*, peroxisome proliferator-activated receptor gamma; *shp*, small heterodimer partner; *srebp1c*, sterol regulatory element-binding protein 1.
